# The P.I.N.K. Study Approach for Supporting Personalized Risk Assessment and Early Diagnosis of Breast Cancer

**DOI:** 10.3390/ijerph18052456

**Published:** 2021-03-02

**Authors:** Michela Franchini, Stefania Pieroni, Edgardo Montrucchio, Jacopo Nori Cucchiari, Cosimo Di Maggio, Enrico Cassano, Brunella Di Nubila, Gian Marco Giuseppetti, Alberto Nicolucci, Gianfranco Scaperrotta, Paolo Belli, Sonia Santicchia, Sabrina Molinaro, on behalf of the PINK Consortium

**Affiliations:** 1 Institute of Clinical Physiology, National Research Council, 56124 Pisa, Italy; michela.franchini@ifc.cnr.it (M.F.); stefania.pieroni@ifc.cnr.it (S.P.); 2 Senologica SrL, 19124 La Spezia, Italy; emontrucchio@gmail.com; 3 Breast Unit, Azienda Ospedaliera Universitaria Careggi, 50139 Firenze, Italy; norij@aou-careggi.toscana.it; 4 Studimed Cadorna Srl, 35123 Padova, Italy; cdimaggio@sirm.org; 5 Istituto Europeo di Oncologia, 20141 Milano, Italy; enrico.cassano@ieo.it (E.C.); brunella.dinubila@ieo.it (B.D.N.); 6 Department of Radiology, Azienda Ospedaliera Universitaria Ancona, 60030 Ancona, Italy; giuseppettigianmarco@gmail.com; 7 Studi Michelangelo SrL, 50129 Firenze, Italy; a.nicolucci@studiomichelangelo.com; 8 Istituto Tumori, 20133 Milano, Italy; gianfranco.scaperrotta@istitutotumori.mi.it; 9 F. Policlinico Gemelli IRCCS, Università Cattolica del Sacro Cuore, 00168 Roma, Italy; paolo.belli@unicatt.it; 10 AUSL della Romagna Centro di Prevenzione Oncologica, 47923 Rimini, Italy; sonia.santicchia@auslromagna.it

**Keywords:** breast cancer, early diagnosis, integrated imaging techniques, personalized medicine, web based data collection

## Abstract

Breast cancer is a clear example of excellent survival when it is detected and properly treated in the early stage. Currently, screening of this cancer relies on mammography, which may be integrated by new imaging techniques for more exhaustive evaluation. The Personalized, Integrated, Network, Knowledge (P.I.N.K.) study is a longitudinal multicentric study involving several diagnostic centres across Italy, co-ordinated by the Italian National Research Council and co-funded by the Umberto Veronesi Foundation. Aim of the study is to evaluate the increased diagnostic accuracy in detecting cancers obtained with different combinations of imaging technologies, and find the most effective diagnostic pathway matching the characteristics of an individual patient. The study foresees the enrolment of 50,000 women over the age of 40 years presenting for breast examination and providing informed consent to data handling. So far, the 15 participating centres across Italy have recruited a total of 22,848 patients. Based on the analyses of the first 175 histopathological-proven breast cancers, mammographic sensitivity was estimated to be 61.7% (*n* = 108 cancers), whereas diagnostic accuracy increased by 35.5% (*n* = 44 cancers) when mammography was integrated with other imaging modalities (ultrasound and/or digital breast tomosynthesis). Increase was mainly determined by ultrasound alone. Given the ongoing data collection and recruitment, the number of cancers detected is too low to allow any further in-depth analysis to explore links to patient characteristics. Past studies show that the uniform approach of population screening guidelines should be revised in favour of more personalised regimens, where known standards are integrated by imaging techniques most suitable for the individual’s characteristics. With the ultimate goal of identifying early breast cancer detection strategies, our preliminary results suggest that integrated diagnostic approach could lead to a paradigm shift from an age-based regimen toward more specific and effective risk-based personalised screening regimens, in order to reduce mortality from breast cancer.

## 1. Introduction

Breast cancer (BC) treated in the early stage is a clear example of excellent survival and closely relies on early screening [1].

Traditional BC screening is based on the mammography technique, used since the 1960s [2]. The overall sensitivity of mammography for BC detection is 75–85%, which can decrease to 30–50% in women with dense breast tissue [3].

Although the trials of mammographic screening provide evidence about its effects on reduction in BC mortality [4,5], mammograms have their limits and newer diagnostic techniques have been introduced to increase the diagnosis of early cancer in those patients for whom mammography is less sensitive. In particular, ultrasound scan (US) introduced in the 1970s, digital breast tomosynthesis (DBT) and magnetic resonance imaging (MRI) in the 1990s have greatly improved the ability to recognise very early carcinomas [2].

Sonographically detected cancers are most often invasive tumours, and their detection will not increase the rate of ductal carcinoma in situ (DCIS) seen at mammography. However, hand-held ultrasound is an operator-dependent method and the skill necessary to detect small tumours limits its universal implementation [3].

DBT use is associated both with an increase in cancer detection rate and a recall rates reduction [6,7,8]. Moreover, DBT in combination with standard digital mammography raises invasive cancer detection by more than a third compared to mammography alone and decreases false positives by 15% [8].

A recent prospective trial, comparing adjunct screening with DBT or US in women with mammography-negative dense breasts, estimated an additional detection rate of 4.9/1000 for ultrasound vs. 2.8/1000 for tomosynthesis. Moreover, the trial’s results showed that the additional false-positive recall was 0.30% for tomosynthesis vs. 1.0% for ultrasound [9].

MRI has been recommended for women with a > 20% lifetime risk of BC [10] although it requires the use of intravenous gadolinium which is a toxic compound in case of patients with renal dysfunction [11] and it is 5–10 times more expensive than the screening mammography cost [12,13,14,15,16]. MRI has a very high true positive rate, a callback rate of 8–17% and a relatively low positive biopsy rate of 20–40% [17].

Screening programmes for a range of conditions focus on increasing the incidence of early-stage detected cancer and decreasing the incidence of late-stage detected cancer [17,18]. Effective screening programmes can deliver significant public and individual health benefits [19] but they can also lead to harms as over-diagnosis, more treatment than necessary (i.e., over-treatment) and no reductions in mortality [1].

The Euroscreen Working Group calculated a summary estimate of over-diagnosis of 6.5% (range, 1% to 10%) on the basis of data from studies in Europe adjusted for both lead time and contemporaneous trends in incidence [20].

The over-diagnosis associated with the BC screening seems to be mainly related to the DCIS [21].

The long-term risks of invasive BC and of death from BC after DCIS, detected by screening, are still poorly understood. More information is needed on how the incidence of invasive BCs varies with the characteristics of the patient, the tumour-related factors (DCIS tumour size, grade, laterality, oestrogen receptor status) and treatments (type of surgery, radiotherapy, endocrine treatment). A recent study by Mannu and colleagues [22] shows that the women with DCIS detected by screening had rates of both invasive BC and death from BC that were over twofold those of the general population, and the increases lasted until at least 20 years after diagnosis.

Each screening programme has different benefits and harm, and the balance between these not only depends on the available options for BC screening but also on other contextual factors, such as the features and the risk profiles of the invited population [17], the optimal surveillance timing [23] and the prevalence of the different breast tumours in the screened population [19].

Defining how often women should undergo mammography screening remains a controversial issue. The most recent recommendations from the European Commission Initiative on BC (ECIBC) [23] suggest less frequent surveillance among asymptomatic women—e.g., for asymptomatic women aged 45 to 49 with an average risk of BC, the ECIBC suggests either triennial or biennial mammograms over annual screening in the context of an organised screening program. Based on tumour biology, some studies in the United States have argued that screening intervals should be shorter for younger women, whereas less frequent screening may be sufficient for women 50 years or older. New Randomised Clinical Trials (RCTs) comparing screening mammography intervals with mortality endpoints are impractical; thus, today screening interval guidelines must rely on observational data and modelling [24].

A study of a prospective cohort from 1996 to 2012, of 15,440 women ages 40 to 85 years with BC diagnosed within 1 year of an annual or within 2 years of a biennial screening mammogram, showed that menopausal status may be more important than age when considering BC screening intervals. [24].

A recent study, based on 168 interval BC patients and 498 matched control subjects, shows that triaging women for breast screening might be substantially improved by considering characteristics as breast density, BMI and other measures related to risk, as family history of BC [25]. Moreover, biological characteristics of breast tumours are highly heterogeneous. Although indolent cancers with likelihood of better outcomes are detected easily by screening mammography, raising the overall incidence of BC, many of the aggressive and lethal forms either go unnoticed on mammograms or develop in the interval between mammograms [26].

Considering all the aspects above, it is recognised that the uniform approach of population screening guidelines is certainly outdated and should be revised in favour of more personalised screening regimens, where screening standards are integrated by imaging modalities most suitable for the individual’s characteristics [1,27,28].

Equipped with knowledge and tools that are more precise, radiologists can select diagnostic approaches (protocols, integrated techniques) based on the patient’s profile that may ensure more targeted diagnostic pathways.

Accordingly, we sought to evaluate the increased diagnostic accuracy in detecting cancers that can be obtained by using different combinations of imaging technologies, and find the most effective diagnostic pathway matching the characteristics of an individual patient, with the ultimate goal of implementing early detection for targeted subgroups.

For this reason, the Personalized, Integrated, Network, Knowledge (P.I.N.K.) study aims to collect data about women with different personal and clinical features, who have undergone at least one periodic breast examination by their own free will, during the study period.

The P.I.N.K. study design is based on a cycle of consensus meetings between expert radiologists and epidemiologists. The primary objective is to compare the diagnostic performance of integrated diagnostic pathways in the early detection of BC in women and quantify the gained diagnostic accuracy. Specifically, the comparison takes into consideration mammography (Mx), US and DBT.

The final aim is to estimate the gained diagnostic accuracy considering each woman’s profile built on personal features (age and other non-modifiable risk factors and breast density) and past/current lifestyles and treatments.

Secondary objectives include: assessing the rate of false positivity and evaluating the most appropriate frequency for surveillance imaging for each risk profile. In order to do so, the study creates a dynamic network between Italian diagnostic centres performing clinical BC diagnosis, and structures a common database containing standardised clinical data collected by means of a specifically developed centralised web platform. The database is enriched with data collected using self-administered questionnaires investigating women risk factors, family history and lifestyle in order to create a comprehensive set of information thus enabling the definition of personalised risk profiles.

The current limited number of cancers verified through the anatomic pathology reports and the partial linkage between the questionnaire and the clinical data not yet allow to quantify the gain diagnostic accuracy by risk profile. So, this paper describes: (1) the P.I.N.K. study design and main features regarding ethical aspects, study population, adopted diagnostic protocol, data collection methodologies and structures; (2) P.I.N.K. current status and initial accomplished results about the pooled additional BC detection rate compared to the mammographic findings alone; (3) its current strengths, limitations and planned evolution.

## 2. Methods

### 2.1. Ethical Considerations

The present study was submitted and approved by the ethical committees of each participating centre. The main approval was provided by the Regional Ethics Committee for Clinical Trials of the Tuscany Region CEAVNO (Prot n 9047 on 19th of February 2018). The CEAVNO ethical committee is responsible for the University Hospital of Pisa, the coordinator centre for the recruitment activity.

The study is being conducted in line with the principles set out in the original Declaration of Helsinki and later amendments. Informed consent has been obtained by all participants and data are handled accordingly.

### 2.2. Study Design and Population

The P.I.N.K. study (Personalized, Integrated, Network, Knowledge study) is an ongoing five-year longitudinal multicentric study, started in October 2017, aiming to recruit 50,000 women of the age of 40 years and above, presenting spontaneously for routine breast examination at several public or private diagnostic centres across Italy.

Researchers involved in the study did not impose more specific limits on the age groups of women to be recruited, as P.I.N.K. is not an organised screening program. Actually, P.I.N.K. aims to integrate all available information on a large number of women with different personal and clinical characteristics, who have undergone periodic breast exams of their own free will.

The study is coordinated by the Italian National Research Council (CNR) and co-funded by Umberto Veronesi Foundation.

The only inclusion criterion for patients is an informed consent to share personal medical data collected during their routine breast clinical examination, whereas having a breast implant, being pregnant or breastfeeding represent criteria for exclusion.

**Figure 1:**
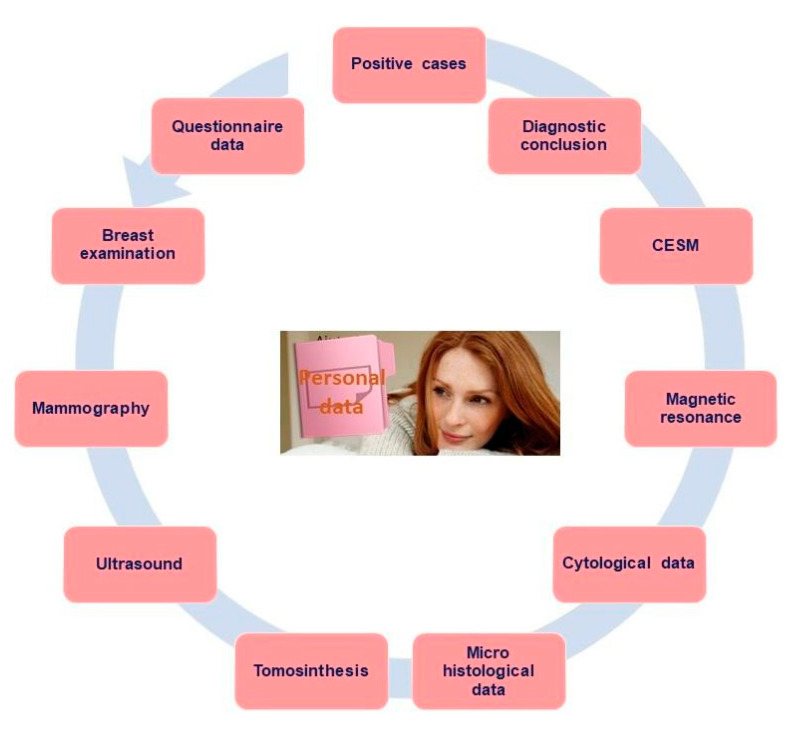
Overview of the Personalized, Integrated, Network, Knowledge study (P.I.N.K.) diagnostic data types.

The study design was jointly decided by the group of radiologists and epidemiologists during the consensus conferences, based on critical appraisal of the most relevant scientific evidence in terms of differences among diagnostic methods available and the modalities adopted by the participating centres. The consensus process delivered a set of core longitudinal diagnostic data to be collected, several diagnostic paths for patients, an articulated database and data elaboration system, and a questionnaire on lifestyle and habits (Figure 1).

### 2.3. Diagnostic Protocol

**Figure 2:**
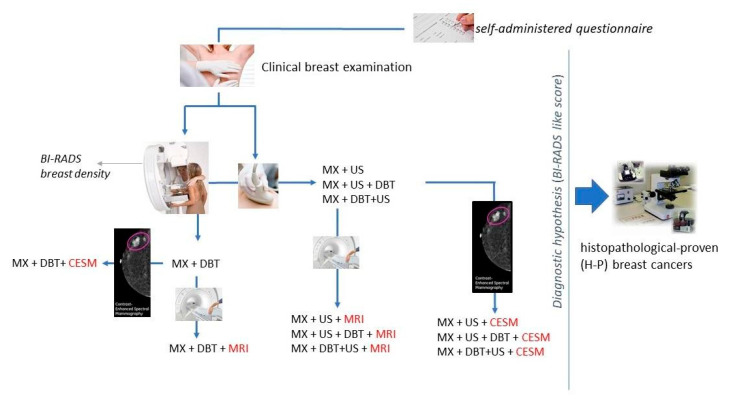
Overview of the P.I.N.K. diagnostic paths.

Women presenting for their visits all received a clinical breast examination and were directed to one of the diagnostic paths foreseen by the P.I.N.K. protocol: (1) mammography + US; (2) mammography + DBT; (3) mammography + US and DBT; (4) mammography + DBT and US. Optionally, magnetic resonance (MRI) and/or contrast enhanced spectral mammography (CESM) are additionally performed determining these additional paths: (5) mammography + US + DBT + MRI; (6) mammography + DBT + US + MRI; (7) mammography + US + DBT + CESM; (8) mammography + DBT + US + CESM (Figure 2).

Imaging diagnostic tools across the P.I.N.K. centres were assessed by the consensus process and considered as presenting similar characteristics in terms of technical features and diagnostic performance.

### 2.4. Data Collection, Measurements and Definitions

#### 2.4.1. Structuring of the Centralised Database Containing Clinical Standardised Data

Relevant BC diagnostic data collected included: personal information, clinical breast examination, MX, DBT, US, MRI, CESM, cytological and micro-histological reports, diagnostic conclusions and cancer cases in-depth description i.e., type of tumour, in situ classification, invasive classification, oestrogen receptor, progesteron receptor, proliferation marker epidermal growth factor, grading, diameter, lymph nodes and the biological and phenotypic characterisation.

The main data structure was designed to host several linked database entities: (a) *breast examination,* storing reason for breast examination, previous tumour presence, first visit, follow-up or subsequent rounds, laterality; (b) *MX,* storing exam type (conventional or synthetic), laterality, density (BI-RADS A-D), location and type and size of the lesions, diagnostic hypothesis; (c) *DBT,* storing laterality, density, number of projections, location and type and size of the lesions, radiodensity of the plane of the lesions, flag indicating if DBT has been done after mammography or after mammography and US, diagnostic hypothesis; (d) *US,* storing laterality, density, location and shape and size of the lesions, flag indicating if US has been done after mammography or after mammography and DBT, diagnostic hypothesis; (e) *Cytological data,* storing diagnostic method originating the cytological exam, diagnosis; (f) *Micro histological data,* storing diagnostic method originating the micro histological exam, method of execution, diagnosis; (g) *MRI* and (h) *CESM,* storing laterality, background, contrast medium, lesion type, kinetic analysis, enhancement type, internal mass enhancement, non-mass enhancement spatial distribution, mass, margins, focus, location, size, diagnostic hypothesis; (i) *Diagnostic conclusion* storing indications for the women, radiologist conclusion; (j) *Cases identification* storing histological tumour type, in situ tumour classification, invasive tumour classification, estrogen receptor (ER), progesteron receptor (PR), proliferation marker, ki67 index, human epidermal growth factor receptor (HER2) grading, diameter, lymph nodes.

The system then automatically classifies the tumour as: (1) LUMINAL A if ER positive & PR positive & HER2 negative & Ki-67 low; (2) LUMINAL B\HER2 NEGATIVE if ER positive & PR positive & HER2 negative & Ki-67 high; (3) LUMINAL B\HER2 POS if ER positive & PR positive & HER2 positive; (4) TRIPLE-NEGATIVE if ER negative & PR negative & HER2 negative; (5) HER2 POS NON-LUMINAL if ER negative & PR negative & HER2 positive.

Each enrolled woman is identified through a unique numeric code which is the only known woman’s identifier (WID). All the data structures store the women identification number, the date and time of the exam and the code of the radiologist performing the data entry. These paths have been implemented in a web platform developed according to the personalised medicine approach; each centre has its own access credentials and each clinician has his own password to access the IT platform, in order to collect clinical data securely.

Clinical data acquisition was standardised using a common data dictionary and a centralised database, implemented within a web platform.

#### 2.4.2. Self-Administered Questionnaire

Women are invited to fill a structured self-administered questionnaire investigating her risk factors, family history and lifestyle through a number of items organised into four main sections: (a) social characteristics such as marital status, education level, job situation; (b) anamnesis (age of menarche and menopause, hormonal therapies, birth control pill, pregnancy and breastfeeding, assisted fertilisation); (c) lifestyle, eating habits, physical exercise, smoking habits and alcohol consumption; (d) the family anamnesis detecting the presence of tumours among first degree relatives.

Each completed questionnaire includes the same WID that identifies every single P.I.N.K. woman along the study. Diagnostic centres participating in the study collect the completed questionnaires and return them to the CNR, where the questionnaires are processed with optical reading technique. At each subsequent access by the same woman, the clinical information is updated by the radiologist directly on the web platform, while the contextual information is provided through the self-administration of a short follow-up questionnaire.

#### 2.4.3. Integration of Clinical Data with the Questionnaire Information

**Figure 3:**
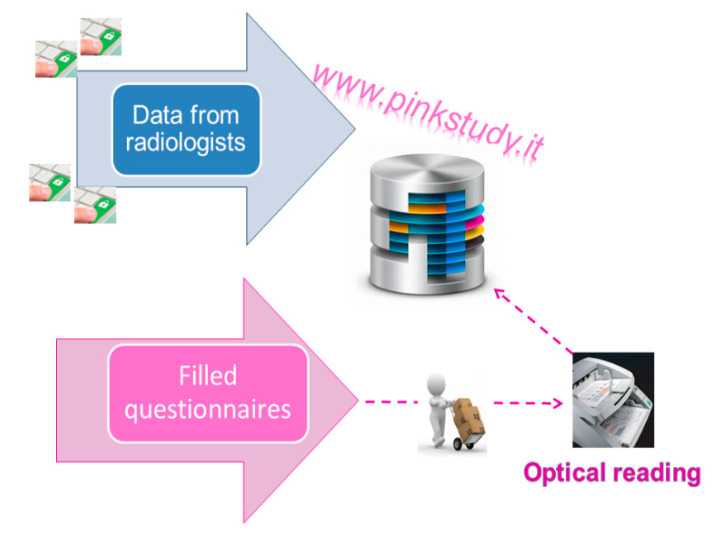
The P.I.N.K. data flow.

The questionnaire digital archives resulting from the optical reading are integrated with the clinical data entered in the web platform: these sources of data are linked through the WID. The data are saved on a centralised relational database (db), hosted by CNR and implemented in MySQL, an open source SQL database management system. The P.I.N.K. dataflow is shown in Figure 3.

#### 2.4.4. Assessment of Added Diagnostic Accuracy Yielded by Integrated Imaging Tests Compared to Mammographic Assessment Alone

The P.I.N.K. protocol foresees that each woman receives a routine breast clinic examination and a MX plus US and/or DBT in the same appointment. The 2D mammographic images are obtained in two standard planes (mediolateral oblique and craniocaudal) using a full-field DM system (FFDM). Combination of 2D and tomosynthesis examinations are acquired under a single compression using a tomosynthesis system. US examinations are performed using a broadband linear transducer with frequency higher than 15 MHz and a digital US unit of last generation.

By default, women are further monitored on a yearly basis (different subsequent rounds in time). In case of clinical suspicion, the radiologist may recommend a lower frequency.

Findings are classified into different levels according to “BI-RADS like” scores (1 to 5), as conventionally agreed during the P.I.N.K. radiologist’s consensus meetings: (1) negative, (2) benign lesion, (3) probably benign lesion, (4) lesions as probably malignant, (5a) positive unifocal, (5b) positive multifocal, (5c) positive multicentric and (5d) positive diffuse. In the event a breast abnormality (BI-RADS greater than 3 in mammography alone or in one of other methodologies provided in the diagnostic pathways) is detected during the examination, the patient is referred for further investigations (histological or cytological testing) for validating the radiologists’ conclusions.

The P.I.N.K. data are periodically extracted to allow statistical-epidemiological analysis where the additional number of cancers detected are used for estimating the additional detection rate. The extraction procedure generates a dataset containing one row for each woman and as many columns as the variables collected during data entry, at baseline, follow-up and subsequent screen rounds.

Starting from participants with histopathological-proven BC, we compare the diagnostic hypothesis of absence of cancer (BI-RADS 1), of benign lesion (BI-RADS 2) or of probably benign lesion (BI-RADS 3) by mammograms, with the diagnostic hypothesis of cancer by other imaging techniques within each diagnostic pathway: (a) mammography and US; (b) mammography and DBT; (c) mammography, DBT and US; (d) mammography, US and DBT. The additional detection rate is estimated for each of the different diagnostic pathways and diagnostics techniques.

The weight of the additional detection rate for the pathways including MRI (high-field, 1.5-3 Tesla) and CESM, recently added to the original study protocol, will also be analysed once collected data, by using these pathways, will be sufficiently robust.

### 2.5. Verification of Diagnostic Concordance among the Different Imaging Methods

Diagnostic concordance was estimated considering the number of identified cases: BI-RADS levels for each different imaging techniques are compared with the histological, cytological or post-surgery results, considered as the standard reference. All the data supporting the concordance estimate are available on the dedicated web platform.

## 3. Results

**Figure 4:**
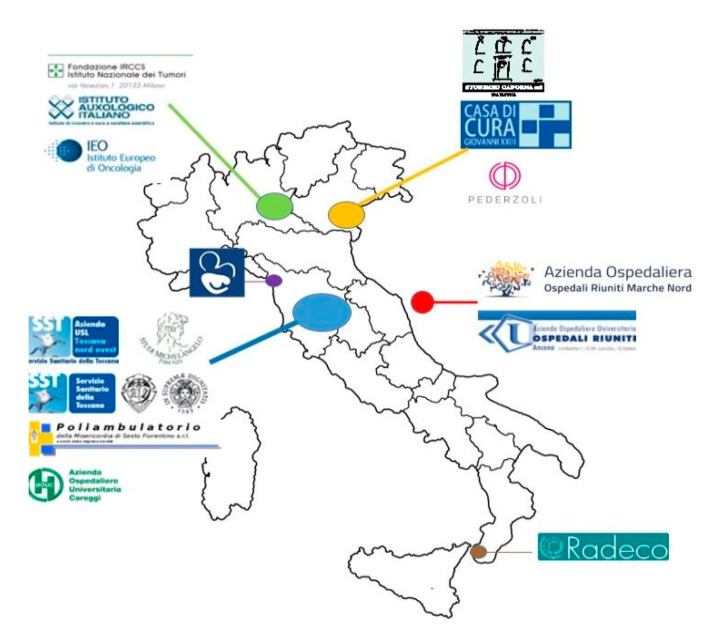
Geographical distribution of 15 P.I.N.K. participating centres. Data at 22 September 2020.

The study currently involves a total of 15 participating centres across Italy, including public medical centres/public hospitals, private medical centres/private hospitals, and Scientific Institute for Research, Hospitalization and Healthcare (IRCCS) (Figure 4).

Dividing by type of healthcare facility, the public medical centres/public hospitals include Azienda Ospedaliera Universitaria Santa Chiara in Pisa, Azienda Ospedaliera Universitaria Careggi in Florence, Azienda USL Toscana Nord Ovest in Massa, AOU Ospedali Riuniti in Ancona, Azienda Ospedaliera Marche Nord in Pesaro. For the private medical centres/private hospitals: Ospedale P. Pederzoli in Peschiera del Garda, Casa di Cura Giovanni XXIII in Treviso, Radeco Srl in Catania, Senologica Srl in La Spezia, Studio Michelangelo in Florence, Misericordia di Sesto Fiorentino SrL in Florence, and STUDIMED Cadorna in Padova. Among the scientific Institute for Research, Hospitalization and Healthcare (IRCCS): Fondazione Istituto Nazionale dei Tumori in Milan, Istituto Auxologico Italiano Ospedale S. Luca in Milan, and Istituto Europeo Oncologia in Milan.

Among these, 10 participating centres started active recruitment in April 2018, four centres in 2019, and one centre in May 2020, recruiting an overall of 22,848 women (Table 1).

The other five centres, one in the northern area, one in the centre, and three in the south are still following the ethical approval process and the administrative pathway.

### 3.1. Population Characteristics

Most participants were asymptomatic (98.2%) and with no previous cancers (94.2%). Mean age was 54.7± 9.8 years. BI-RADS breast density on mammography of most women was either B (34.6%) or C (37.0%). Table 2 reports the percentage distribution of participants by age and breast density (Table 2).

### 3.2. Gained Detection Rate

Considering the first 175 P.I.N.K. women with histopathological diagnosis of BC, we calculated a MX sensitivity of 61.7% (95%CI: 54.1–69.0%) referred to the 108 cancers detected by mammography.

**Figure 5:**
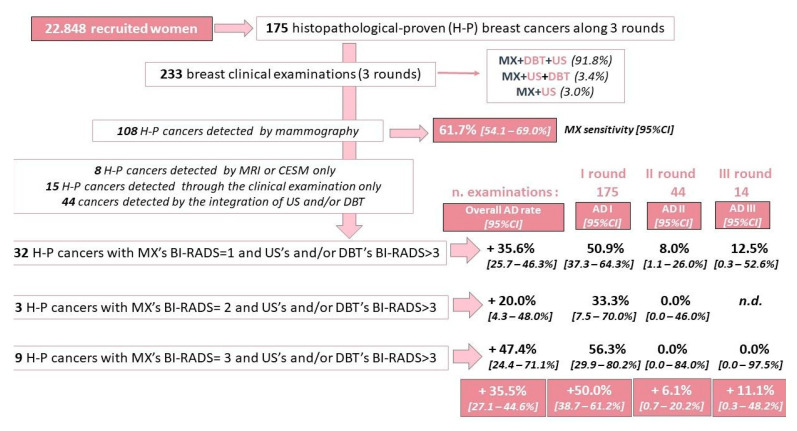
Preliminary result synthesis—Histopathological-proven breast cancer (BC) extracted in October 2020.

The 175 BC women have undergone a total of 233 examinations: all 175 women completed the first examination (first round), 44/175 have undergone a second examination (second round) and 14/44 completed the third examination (third round). Preliminary estimation of the pooled additional detection rate was obtained by comparing the mammographic findings (BI-RADS scores 1–3) with those obtained by the other diagnostic imaging techniques, yielding an additional detection rate of 35.6% (95%CI: 25.7–46.3%) among women with a negative mammographic hypothesis (BI-RADS 1; *n* = 90 examinations considering all the three rounds), 20.0% (95%CI: 4.3–48.0%) among women having a mammographic diagnostic hypothesis of benign lesion (BI-RADS 2; *n* = 15 examinations considering all the three rounds), and 47.4% (95%CI: 24.4–71.1%) among women with a diagnostic hypothesis of probably benign lesion (BI-RADS 3; *n* = 19 examinations considering all the three rounds). Overall additional detection rate was 35.5% (95%CI: 27.1–44.6%) (Figure 5).

Among the first 175 cancers, women following the “MX, DBT and US” pathway totalled 91.8% (214 out of 233 clinical examinations along three subsequent rounds), which was associated with an overall additional detection rate of this pathway of 36.0% (95%CI: 27.2–45.4%). This additional rate was mainly determined by the additive cases detected by US alone (+18 cancers) identified as BI-RADS 4-lesion of uncertain malignant potential (+11 cancers) and BI-RADS 5-positive unifocal (+7 cancers).

**Figure 6:**
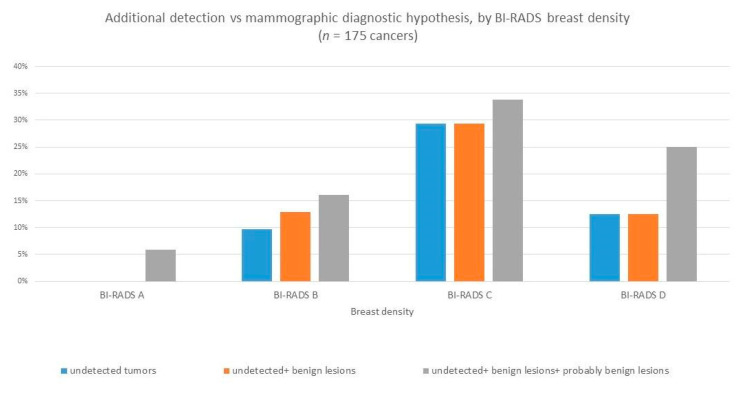
Additional detection rate by breast density.

**Figure 7:**
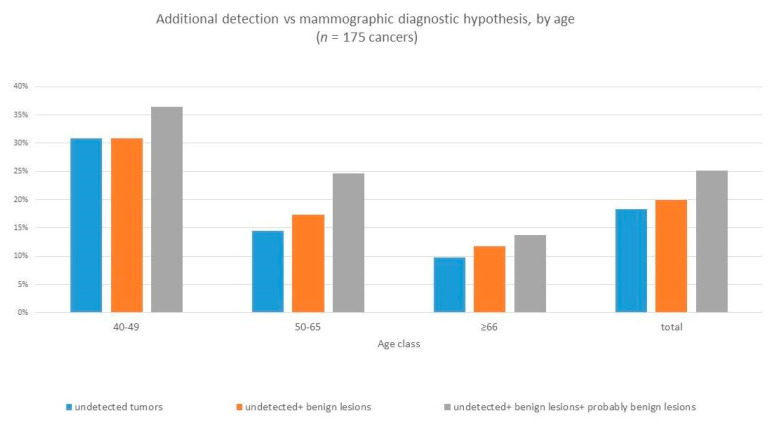
Additional detection rate by age class.

The higher cancer detection rates of the different diagnostic imaging techniques appeared to be associated to the woman’s breast density (Figure 6) and age (Figure 7), with the greatest additional detection rate resulting in women in the younger age group (40–49 years of age) and with denser breast tissue (BI-RADS C and BI-RADS D).

Regarding the frequency of breast examination, the time between examinations was 12 months for 65.5% (*n* = 38) of all examinations (*n* = 58); between 13 and 18 months for 29.3% (*n* = 17) and over 18 months for 5.2%.

The current limited number of cancers verified through the anatomic pathology reports provides partial results and prevents further in-depth analyses linked to the individual profiles identified by the data from the P.I.N.K. questionnaires.

## 4. Discussion

Medical imaging has many advantages including real time monitoring, accessibility without tissue destruction, minimal or no invasiveness and can work over wide ranges of time and size scales involved in biological and pathological processes [29]. Concerning the wide range of usage along the cancer disease process including diagnosis, staging, therapy planning, monitoring and surveillance, imaging is considered as an essential component of clinical cancer protocols [30]. Although mammography is the current standard breast screening technique, it is less effective for subjects under 40 years old and dense breasts, partly less sensitive to small tumours and providing limited indication of eventual disease outcome [31]. Therefore, the integration with new imaging modalities may be appropriate, in particular for women whose individual characteristics, risk factors and life habits pose them at higher risk [3,32].

Many studies stated that early-stage (Stage I/II) diagnosed BCs have better prognosis in terms of 5-year survival rate (85–98%) while late diagnosed BCs have poor 5-year survival rate (30–70%) [33].

Moreover, using the incidence rates of fatal cancers, Tabar and colleagues [34] directly compared cancers diagnosed during the study period among women who did and did not participate in mammography screening. Tabar found a lower incidence of cancers that were fatal at either 10 years (60%) or 20 years (47%) in the participating women. He also concluded that this difference is attributable to earlier detection and earlier treatment, among women participating in mammography screening [34].

Not least, the stage of disease at diagnosis is an important predictor of treatment costs. Treatment for more advanced disease is often more intensive or invasive than treatment for the earlier stages [35]. As a result, a more advanced stage tends to be associated with more resource utilisation in addition to poorer health outcomes [36]. A recent review estimated that the mean treatment costs of stages II, III and IV BCs are respectively 32%, 95% and 109% higher than the ones of stage I [37].

Preliminary results from our study, referring to the first 175 women with histopathological diagnosis of BC, show that the combination of different imaging techniques improves the BC detection rate by 35.5% (95%CI: 27.1–44.6%). The additional contribution is mainly determined by the additive cases detected by the US alone.

Moreover, we found that the breast examination frequency was annual for 65.5% of examinations, while the 34.5% of patients underwent tests less frequently. This data could be more informative once we will progress with the analysis including other aspects as the cancer phenotypes and the individual profile of risk.

### 4.1. Study Outcomes and Next Developments

The P.I.N.K. study is half-way and the anatomic pathology report requested for the suspected tumours is still not available for many patients, so these initial results need to be reviewed once the data will be complete. Further analysis will be performed once the examinations of consecutive rounds are more numerous, the recruitment is ended and the self-administered questionnaire data is fully linked with the clinical data.

At that point it will be possible to evaluate correlations between the gained diagnostic accuracy and each woman’s profile built on personal features (age and other non-modifiable risk factors, breast density) and past/current lifestyles or treatments. Moreover, the personalised approach could also suggest the most appropriate frequency for surveillance imaging.

Finally, the P.I.N.K. consortium has acknowledged the large amount of additional data needed to identify specific patterns of cancer onset compared to those routinely collected in the original protocol. Therefore, starting in 2021, the P.I.N.K. study will carry on with future investigations. In particular, other data dimensions such as lifestyle, nutrition habits, imaging biomarkers will be collected. A development hypothesis is to integrate clinical data (from the web platform) with imaging data and data collected through more in depth lifestyle questionnaires (nutrition and physical activity). The subsequent application of advanced data analysis methods (e.g., image analysis techniques, Big Data analytics and machine learning) enables to pursue different scenarios that cover the entire pathological process of BC, from prevention to monitoring of disease evolution.

### 4.2. Strength and Limitations

The population included in the present study is different from the one who attends a population-based screening. The main differences are in terms of women’s propensity of undergoing periodic breast examination on a voluntary basis, age classes, adopted diagnostic imaging techniques and frequency of controls. These differences do not limit the generalisability of our study results, but they limit the comparability with population-based screening results.

However, this study presents valuable epidemiological data based on a broad-ranged recruitment plan, involving a vast network of high-quality care/diagnostic centres and radiologist investigators with consolidated experience in clinical breast imaging who perform over 1500 diagnostic pathways per year.

Diagnostic procedures and technologies employed have all been assessed for performance quality and standardised procedures through the consensus process. Individual data from self-administered questionnaires, linked with clinical data through the WID, ensure the data integrity and completeness. The WID use ensures pseudonymisation of personal data and allows respecting for data security requirements.

Given that data collection is still ongoing, we are obviously prevented from providing full results: the recruitment will continue in the next year and anatomic pathology reports are still under submission in the web platform. Moreover, part of the questionnaires filled by the women are not yet linked to the rest of information stored in the P.I.N.K. database: this is in fact a backend scheduled procedure conditioned by the return times by the centres and by the processing times through optical reading, mediated by the human operator. Moreover, once the P.I.N.K. database is completed, the results of epidemiological analysis could also allow to estimate the risk of false-positive results, not available at the current stage of the analysis and it could strengthen the current population-based screening toward a more personalised approach.

## 5. Conclusions

The P.I.N.K. study has been designed for supporting the hypothesis that, the more accurate the identification of each woman’s risk profile, the better the capability of performing integrated diagnostic processes targeted for the woman’s features and improving earlier diagnosis. This could modify the natural history of the disease, with the final aim of reducing mortality.

Our work, focusing on the role of women’ features and risk profiles in the identification of the most appropriate diagnostic pathways for BC early detection, could balance the different benefits and harm within a screening programme. This innovative approach could lead to a paradigm shift from an age-based regimen towards more specific and effective risk-based personalised screening regimens, involving patients, providers, facilities, health care systems and regional/national organisations.

## Figures and Tables

**Table 1:** P.I.N.K. recruitment status by area. Data at 22 September 2020.

Geographic Area	Centre Typology	Recruited Women
**NORTH**	Public medical centre/hospital	4593
	Private medical centre/hospital	6738
	IRCCS	2607
***Subtotal for North Italy***		13,938
**CENTRUM**	Public medical centre/hospital	2387
	Private medical centre/hospital	6305
***Subtotal for Centrum Italy***		8692
**SOUTH**	Private medical centre/hospital	218
***Subtotal for South Italy***		218
	**Total**	22,848

**Table 2:** Distribution of patients by age group and breast density score (BI-RADS classification A-D). N = 22,848.

Age Class	Density	Women	Percentages by Age Class	Age Class	Density	Women	Percentages by Age Class
40–44	A	192	6.6%	65–69	A	498	25.0%
	B	678	23.3%		B	862	43.3%
	C	1248	42.9%		C	536	26.9%
	D	793	27.2%		D	94	4.7%
45–49	A	296	6.4%	70–74	A	416	28.4%
	B	1179	25.6%		B	641	43.7%
	C	2040	44.2%		C	356	24.3%
	D	1096	23.8%		D	54	3.7%
50–54	A	440	9.6%	75–79	A	230	29.9%
	B	1572	34.2%		B	320	41.6%
	C	1841	40.0%		C	189	24.6%
	D	749	16.3%		D	30	3.9%
55–59	A	529	14.7%	80–84	A	67	27.9%
	B	1396	38.7%		B	120	50.0%
	C	1357	37.6%		C	48	20.0%
	D	325	9.0%		D	5	2.1%
60–64	A	485	18.5%	>85	A	11	33.3%
	B	1133	43.3%		B	10	30.3%
	C	836	31.9%		C	12	36.4%
	D	164	6.3%		D	0	0.0%

## Data Availability

We provide the up to date version of the baseline and follow-up questionnaires, the P.I.N.K. data structure and the informative materials.
